# Transcriptome analysis provides novel insights into the soil amendments induced response in continuously cropped *Codonopsis tangshen*

**DOI:** 10.3389/fpls.2022.972804

**Published:** 2022-08-12

**Authors:** Wuxian Zhou, Xiaogang Jiang, Xuhui Tan, Darong Li, Hua Wang, Jinwen You, Xiaoling Li, Meide Zhang

**Affiliations:** Key Laboratory of Biology and Cultivation of Herb Medicine, Ministry of Agriculture and Rural Affairs, Institute of Chinese Herbal Medicines, Hubei Academy of Agricultural Sciences, Enshi, China

**Keywords:** *Codonopsis tangshen*, continuous cropping, soil remediation, RNA-Seq, metabolic pathway, qRT-PCR

## Abstract

*Codonopsis tangshen* Oliv (*C. tangshen*) is an important Chinese traditional medicinal plant with various health benefits. However, the growth of *C. tangshen* are seriously affected by continuous cropping, which led to the decrease of the yield and quality. A field experiment was conducted to learn the effects of soil amendments on the growth of *C. tangshen* under continuous cropping condition, and the biological events which occurred at molecular level were investigated. The results indicated that the content of chlorophyll a (Chl a), chlorophyll b (Chl b), and carotenoid (Car) was significantly higher in SCPM (silicon-calcium-potassium-magnesium fertilizer), SCPMA (SCPM combined with azoxystrobin) and SCPMAOM (SCPM combined with azoxystrobin and organic manure) treatments. Moreover, the yield and the levels of alkaloid, polysaccharide, flavone and total protein in the treatments of SCPM, SCPMA and SCPMAOM were significantly higher than those in the control, and these indexes were all highest in the SCPMAOM treatment. RNA-sequencing (RNA-Seq) is an economical and efficient method to obtain genetic information for species with or without available genome data. In this study, RNA-Seq was performed to understand how continuously cropped *C. tangshen* responded to the soil amendments at the transcriptome level. The number of differentially expressed genes (DEGs) were as follows: CK vs. SCPM (719 up- and 1456 down-), CK vs. SCPMA (1302 up- and 1748 down-), CK vs. SCPMAOM (1274 up- and 1678 down-). The soil amendments affected the growth of *C. tangshen* mainly by regulating the genes involved in pathways of ‘photosynthesis,’ ‘plant hormone signal transduction,’ ‘biosynthesis of unsaturated fatty acids,’ ‘phenylpropanoid biosynthesis,’ and ‘starch and sucrose metabolism,’ etc. qRT-PCR was performed to validate the expressions of 10 target genes such as *CP26*, *PsaF*, and *POX*, etc., which verified the reliability of RNA-Seq results. Overall, this study revealed the roles and underlying mechanisms of the soil amendments in regulating the growth of continuously cropped *C. tangshen* at transcriptome level. These findings are beneficial for improving the continuous cropping tolerance and may be valuable for future genetic improvement of *C. tangshen*.

## Introduction

*Codonopsis tangshen* Oliv., a perennial herbaceous species, is an important medicinal herb and was highly valued in traditional Chinese medicine (Xu et al., 2021). The dried root of *C. tangshen* mainly contains lobetyolin, polysaccharides and saponin, and is usually used for curing spleen deficiency, hypertensive disease and hypoglycemic disease (Wu et al., 2020; Xu et al., 2021). Moreover, *C. tangshen* is not only a medicinal herb, but also a food material, and sometimes is used as a succedaneum for *Panax ginseng* due to its similar medicinal value and low price (Tsai and Lin, 2008). However, our previous researches revealed that continuous cropping obstacle (also known as replant disease) was a significant limiting factor for the growth and development of *C. tangshen* (He et al., 2019; Zhang et al., 2021), and thus seriously affected the production and led to the shortage of *C. tangshen* supply. Consequently, *C. tangshen* grown in the continuously cropped land showed smaller leaves, thinner stems, lower biomass, and became more rigid than those grown in the non-continuously cropped land (He et al., 2019). In addition, continuous cropping led to a decrease in rhizospheric soil bacterial abundance, and altered the microbial community structure, thereby resulting in a poor growth of *C. tangshen* under continuous cropping system (Zhang et al., 2021). Therefore, the studies on alleviating negative effects on *C. tangshen* growth induced by continuous cropping were of great significance in agricultural production.

There are lots of methods to eliminate the continuous cropping obstacle of crops, and application of soil amendments is the most widely used measure in agricultural practice. Silicon–calcium–potassium–magnesium fertilizer (SCPM) is an alkaline fertilizer formed by calcination of phosphogypsum and potassium feldspar at high temperature, and is widely used in the remediation of acidified paddy soil (Ji et al., 2019). Organic manure (OM) has been widely used in agriculture to improve the soil environment. OM combined with biochar amendments could promote the growth of Xinjiang cotton by ameliorating the soil in continuous cropping system (Zhang et al., 2020). Azoxystrobin (AZO) is a fungicide which inhibits fungi by disrupting ATP production in fungal mitochondria (Sun et al., 2018). Wang et al. (2022) found that application of appropriate AZO combined with chloropicrin fumigation could promote the growth and phosphorus amount of ginger rhizomes. RNA-Seq has become an indispensable tool for transcriptome-wide analysis of differential gene expression and differential splicing of mRNAs in the past decade (Stark et al., 2019). Chen et al. (2020) reported the transcriptome change of continuously cropped strawberry in response to a soil amendment and indicated that strawberry plants reallocated defense resources to development when soil amendments alleviated the stress caused by continuous cropping. Lu et al. (2020) characterized the transcriptional response of continuously cropped soybeans to *Funneliformis mosseae* and found that the increased activity of some disease-resistant genes may partly account for the ability of the plants to resist diseases. Our previous study indicated that the soil amendments including OM and SCPM could effectively relieve the continuous cropping obstacles to *C. tangshen* cultivation (Zhou et al., 2021), but the recovery mechanisms underlying this phenomenon is still remain unclear. Based on these limited reports, here we hypothesized that soil amendments might directly affect the transcriptome profiles of continuously cropped plants, and therefore induce physiological and phenotypical variations.

In this study, we performed a transcriptome analysis to learn the soil amendments induced response in continuously cropped *C. tangshen* and explore the biological pathways involved in the soil remediation process. Moreover, we addressed the question of whether and how the increased tolerance of *C. tangshen* was associated with the maintenance of photosynthesis, plant hormone signal transduction, biosynthesis of unsaturated fatty acids, phenylpropanoid biosynthesis, and starch and sucrose metabolism, etc. The present study provided a technical support for improving the tolerance of *C. tangshen* to continuous cropping and laid a foundation for unraveling the molecular mechanisms of soil amendments in regulating the growth of continuously cropped *C. tangshen* at transcriptome level.

## Materials and methods

### Plant materials and experimental design

The seeds of *C. tangshen* were used as experimental materials. The field experiment was conducted in Xintian Village (30°32’16”N, 109°12’45”E, 1738 m above sea level), Banqiao Township, Enshi City, Hubei Province, China. A field previously cultivated with *C. tangshen* for 3 years was selected as experiment plot. This study consisted of four treatments, i.e., (1) CK, conventional fertilization; (2) SCPM, application of 3000 kg/hm^2^ silicon-calcium-potassium-magnesium fertilizer; (3) SCPMA, application of 3000 kg/hm^2^ SCPM combined with azoxystrobin (A) 500 times solution; (4) SCPMAOM, application of 3000 kg/hm^2^ SCPM and 4500 kg/hm^2^ organic manure (OM), combined with azoxystrobin 500 times solution. All the amendments were used following the manufacturer’s recommendations. There were three replicates (plots) for each treatment, and each plot size was 20 m^2^. Ditches were set up between the plots (with an interval of 30 cm) to prevent mutual infiltration of soil amendments. The nitrogen application rate was 346.5 kg.hm^–2^, and m (N): m (P_2_O_5_): m (K_2_O) = 1:0.72:0.65. Urea, calcium magnesium phosphate fertilizer and potassium sulfate were used as nitrogen, phosphorus and potassium fertilizer, respectively. All the treatments maintained the same application rate of nitrogen, phosphorus and potassium by adjusting the ratio of base fertilizer.

On March 20, 2019, all the amendments were applied to the soil, and the fertilizer of 50% nitrogen, 100% phosphorus and 100% potassium were also applied at the same time, then the plots were fully plowed and the *C. tangshen* seeds were sown on April 11, 2019. In addition, the remaining 50% nitrogen fertilizer was applied at the seedling stage (20%) and flowering stage (30%) of *C. tangshen*. Moreover, the leaves of the same part were collected on June 6, 2020, and were separated into two parts. One part was used to measure the physiological indexes such as chlorophyll (Chl), superoxide dismutase (SOD), peroxidase (POD), catalase (CAT), soluble sugar (SS), soluble protein (SP), superoxide anion radical (SAR), and malondialdehyde (MDA). The other part was immediately stored in liquid nitrogen and brought back to the laboratory for transcriptome sequencing. The fresh roots of *C. tangshen* were harvested on September 8, 2020, then washed and dried at 60°C. The quality indexes, such as lobetyolin, polysaccharides and alkaloids were measured.

### Measurement of physiological and biochemical traits

The contents of Chl, MDA and SP, and the activities of antioxidant enzymes such as POD, SOD and CAT, etc, were measured according to the previous method (He et al., 2019). Yield, lobetyolin, polysaccharide, etc., were measured according to our previous study (Zhang et al., 2021).

### RNA extraction and cDNA library construction

Total RNA was extracted from the leaf samples using TRIzol reagent (Invitrogen, New York, CA, United States). RNA samples’ concentration, purity and integrity were detected using the Agilent 2100 Bioanalyzer (Agilent, Palo Alto, CA, United States) to ensure high-quality samples for transcriptome sequencing. About 1 μg RNA per sample was used to construct sequencing library. In brief, mRNA was purified from the total RNA with poly-T oligo-attached magnetic beads. Then, mRNA was interrupted to 200–300 bp by ion interruption. First-strand cDNA was generated using random hexamer primer and M-MuLV Reverse Transcriptase, followed by the synthesis of second-strand cDNA using DNA Polymerase I and RNase H. In total, 12 libraries were generated using NEBNext^®^Ultra™ RNA Library Prep Kit for Illumina^®^ (NEB, Lincoln, NE, United States), following the manufacturer’s recommendations. Then PCR enrichment was carried out using Phusion High-Fidelity DNA polymerase, Universal PCR primers and Index Primer. Subsequently, the fragments about 450 bp in length were selected. Afterward, cDNA libraries were purified and quantified by the Agilent 2100 Bioanalyzer. In the end, the libraries were sequenced using an Illumina Hiseq 6000 platform in Shanghai Personal Biotechnology Co., Ltd (Shanghai, China).

### Bioinformatics analysis

#### Processing and assembling of illumina reads

Firstly, the reads numbers, base numbers, Q20, Q30, GC-content, and N (%) were counted based on raw data. Then, clean reads were obtained by removing low-quality reads and reads containing adapters. Subsequently, based on high quality clean data, transcriptome assembly was performed using the Trinity (Version 2.10.0; Grabherr et al., 2011), then the transcript and unigene were identified and used for further analysis.

#### Gene expression analysis

The read count value of each gene was quantified using RSEM (RNA-Seq by Expectation Maximization) (Li and Dewey, 2011), and FPKM (Fragments Per Kilobase of exon per Million mapped fragments) was used for estimating the gene expression level. Then, differential gene expression analysis of two groups was performed using DESeq2 R package (Version 1.10.1; Love et al., 2014) based on the model of negative binomial distribution. The *P*-values were adjusted to control the false discovery rate with the Benjamini and Hochberg’s method. Genes with an adjusted *p* value < 0.05, | log2 Fold Change| > 1 were taken as differentially expressed gene (DEG).

#### Hierarchical cluster, gene ontology, and kyoto encyclopedia of genes and genomes pathway enrichment analysis

Hierarchical clustering was carried out for DEGs. The FPKM counts for each unigene were two-way clustered using gplots in the R package (R Core Team, 2012). Gene Ontology (GO) enrichment of the differentially expressed genes (DEGs) was analyzed by the top GO R packages (Young et al., 2010). Kyoto encyclopedia of genes and genomes (KEGG) enrichment of DEGs between two groups was analyzed by the R package pathfinder (Ülgen et al., 2019).

### Validation of RNA-seq data by real-time quantitative PCR

Real-time quantitative RT-PCR was performed to validate the RNA-Seq results according to the previous method (Li et al., 2017a). The GAPDH gene was used as a reference gene. The qRT-PCR primers in this study are listed in Supplementary Table 1. The 2^–ΔΔ*CT*^ method was used to determine the relative expression of target genes (Kenneth and Thomas, 2002).

### Statistical analysis

The plant sampling in this study followed the principle of random sampling. The data are presented as the mean of three replications and corresponding standard deviation. The differences between the samples were determined by Duncan’s multiple range test at *p* < 0.05. Pearson correlation analysis was performed to analyze the correlations among samples in different treatments. Statistics and correlation analysis were performed using SPSS v20.0 (SPSS Inc., United States). Principal component analysis (PCA) was performed in Canoco 5.0. Bar charts were plotted using OriginPro 2021. Heatmaps and hierarchical clustering were performed using R package models or TBtools software (Version 1.068).

## Results

### Physiological and biochemical properties

The morphological observation showed that the leaves of *C. tangshen* in SCPM, SCPMA and SCPMAOM treatments were larger and greener than those in the control (Supplementary Figure 1). Physiological and biochemical properties were compared among remediation groups and control group. Compared with the control (CK), the Chl a, Chl b, and Car levels were significantly higher in SCPM, SCPMA and SCPMAOM, and the highest levels of these indexes were occurred in SCPMAOM (Table 1). Specifically, the SCPMAOM treatment increased the Chl a, Chl b and Car levels by 24.5, 23.1, and 23.6%, respectively, compared with the control. The SOD activity and MDA content were significantly down-regulated in the three treatments, compared with the control, and with lowest levels in SCPMAOM. Moreover, the SAR and SS levels were significantly decreased in SCPM, SCPMA and SCPMAOM, while the SP level was significantly increased in these three treatments, compared with the control (Table 2).

**TABLE 1:** Content of chlorophyll and carotenoid in *C. tangshen* leaves in different treatments.

Treatment	Chl *a* (mg.g^–1^)	Chl *b* (mg.g^–1^)	Chl (mg.g^–1^)	Car (mg.g^–1^)	Chl a/Chl b	Chl/Car
CK	1.59 ± 0.07 c	0.39 ± 0.01 c	1.99 ± 0.09 c	0.78 ± 0.04 c	4.05 ± 0.04 a	2.54 ± 0.04 b
SCPM	1.70 ± 0.04 b	0.42 ± 0.03 bc	2.12 ± 0.05 b	0.85 ± 0.02 b	4.06 ± 0.21 a	2.51 ± 0.03 b
SCPMA	1.75 ± 0.02 b	0.43 ± 0.01 b	2.18 ± 0.03 b	0.87 ± 0.03 b	4.08 ± 0.05 a	2.51 ± 0.05 b
SCPMAOM	1.98 ± 0.08 a	0.48 ± 0.01 a	2.46 ± 0.08 a	0.94 ± 0.03 a	4.10 ± 0.13 a	2.62 ± 0.01 a

CK, control; SCPM, 3000 kg/hm^2^ silicon-calcium-potassium-magnesium fertilizer; SCPMA, SCPM + azoxystrobin 500 times solution; SCPMAOM, SCPMA + 4500 kg/hm^2^ organic manure. Chl *a*, Chlorophyll *a*; Chl *b*, Chlorophyll *b*; Chl, Chlorophyll *a* + Chlorophyll *b*; Car, Carotenoid. The data are the mean ± SD (n = 3) and the different letters (a–c) indicate a significant difference at *P* < 0.05 according to Duncan’s test.

**TABLE 2:** Superoxide dismutase (SOD), POD, CAT activities and MDA, SAR, SP, and SS levels in *C. tangshen* leaves in different treatments.

Treatment	SOD (U g^–1^.FW.min^–1^)	POD (U g^–1^.FW.min^–1^)	CAT (U g^–1^.FW.min^–1^)	MDA (mg.g^–1^.FW)	SAR (μ g.g^–1^.FW)	SP (mg.g^–1^.FW)	SS (mg.g^–1^.FW)
CK	539.12 ± 31.07 a	135.91 ± 13.88 a	510.75 ± 21.16 c	2.58 ± 0.06 a	133.73 ± 7.39 a	2.06 ± 0.05 c	7.27 ± 0.19 a
SCPM	470.43 ± 22.41 b	140.14 ± 17.70 a	492.55 ± 21.76 c	2.35 ± 0.06 b	118.96 ± 6.71 b	2.39 ± 0.05 b	7.16 ± 0.06 ab
SCPMA	417.85 ± 44.88 b	146.03 ± 5.38 a	631.53 ± 40.68 a	2.00 ± 0.14 c	112.31 ± 4.70 b	2.46 ± 0.05 ab	7.15 ± 0.09 ab
SCPMAOM	381.69 ± 18.71 c	145.29 ± 4.29 a	598.90 ± 29.71 b	1.94 ± 0.14 c	109.17 ± 3.29 b	2.53 ± 0.04 a	6.94 ± 0.21 b

CK, control; SCPM, 3000 kg/hm^2^ silicon-calcium-potassium-magnesium fertilizer; SCPMA, SCPM + azoxystrobin 500 times solution; SCPMAOM, SCPMA + 4500 kg/hm^2^ organic manure. SOD, Superoxide dismutase; POD, Peroxidase; CAT, Catalase; MDA, Malondialdehyde; SAR, Superoxide anion radical; SP, Soluble protein; SS, Soluble sugar. The data are the mean ± SD (*n* = 3) and the different letters (a–c) indicate a significant difference at *P* < 0.05 according to Duncan’s test.

In this study, important indexes such as yield, lobetyolin, polysaccharide, flavone, alkaloid and total protein were measured to learn the effects of soil amendments on yield and quality of *C. tangshen* under continuous cropping condition (Table 3). The results showed that the yield and alkaloid levels of SCPM, SCPMA, and SCPMAOM treatments were significantly higher than those of the control, and the yield of SCPMAOM treatment (7179.5 kg/hm^2^) was 10 times higher than that of the control (683.8 kg/hm^2^). Meanwhile, the SCPMAOM treatment increased the polysaccharide, flavone and total protein levels by 8.5, 22.8, and 43.0%, compared with the control.

**TABLE 3:** Yield and quality of *C. tangshen* in different treatments.

Treatment	Yield (kg.hm^–2^)	Lobetyolin (mg.kg^–1^)	Polysaccharide (mg.g^–1^)	Flavone (mg.kg^–1^)	Alkaloid (mg.g^–1^)	Total protein (mg.g^–1^)
CK	683.8 ± 85.5 d	2.12 ± 0.11 a	587.72 ± 22.50 b	10.66 ± 0.91 b	0.81 ± 0.08 b	5.58 ± 0.38 c
SCPM	5014.2 ± 215.1 c	2.09 ± 0.12 a	615.48 ± 20.18 ab	12.48 ± 0.91 a	1.18 ± 0.02 a	5.81 ± 0.42 bc
SCPMA	6324.8 ± 256.4 b	2.06 ± 0.11 a	609.79 ± 22.29 ab	12.18 ± 0.53 ab	1.21 ± 0.11 a	6.54 ± 0.44 b
SCPMAOM	7179.5 ± 427.4 a	2.05 ± 0.09 a	637.54 ± 16.43 a	13.09 ± 1.05 a	1.10 ± 0.07 a	7.98 ± 0.39 a

CK, control; SCPM, 3000 kg/hm^2^ silicon-calcium-potassium-magnesium fertilizer; SCPMA, SCPM + azoxystrobin 500 times solution; SCPMAOM, SCPMA + 4500 kg/hm^2^ organic manure. The data are the mean ± SD (*n* = 3) and the different letters (a–d) indicate a significant difference at *P* < 0.05 according to Duncan’s test.

### Transcriptome sequencing and assembly

To comprehensively explore the molecular response of continuously cropped *C. tangshen* to soil remediation. 12 cDNA libraries were constructed from the leaves of *C. tangshen* in different treatments including CK, SCPM, SCPMA, and SCPMAOM. All the libraries were sequenced using the Illumina HiSe 6000 platform. The results of sequence assembly were presented in Table 4. The clean reads of CK, SCPM, SCPMA and SCPMAOM treatments were 38715892, 39901087, 40037621, and 40499491, respectively. In addition, the Q20 values were 97.73, 97.64, 97.61, and 97.74%, and the Q30 values were 93.91, 93.75, 93.68, and 93.98%, for CK, SCPM, SCPMA, and SCPMAOM treatments, respectively, which indicated the high quality of the transcriptome sequencing.

**TABLE 4:** Overview of the sequencing data.

Sample	Raw reads	Clean reads	Clean bases (bp)	Q20 (%)	Q30 (%)
CK_1	43740344	39704800	5955720000	97.69	93.93
CK_2	44559232	40738044	6117399784	97.75	93.94
CK_3	39027538	35704832	5355724800	97.76	93.86
average	42442371	38715892	5809614861	97.73	93.91
SCPM_1	43683572	40358522	6061305494	97.51	93.43
SCPM_2	43402718	40139764	6029340136	97.51	93.49
SCPM_3	42998898	39204976	5889608858	97.90	94.32
average	43361729	39901087	5993418163	97.64	93.75
SCPMA_1	44312486	40517910	6085032390	97.65	93.85
SCPMA_2	43601282	39860814	5987391078	97.45	93.22
SCPMA_3	43389520	39734140	5968209054	97.74	93.97
average	43767763	40037621	6013544174	97.61	93.68
SCPMAOM_1	43047134	39522422	5936372172	97.68	93.84
SCPMAOM_2	44583964	40758010	6120955322	97.81	94.13
SCPMAOM_3	44818612	41218042	6189824784	97.72	93.97
average	44149903	40499491	6082384093	97.74	93.98

CK, control; SCPM, 3000 kg/hm^2^ silicon-calcium-potassium-magnesium fertilizer; SCPMA, SCPM + azoxystrobin 500 times solution; SCPMAOM, SCPMA + 4500 kg/hm^2^ organic manure. Raw reads, the total number of sequenced raw reads; Clean Reads, the number of pair-end Reads in the clean data; Clean bases, total base number in the clean data; Q20 (%), Q20 base percentage in the clean data; Q30 (%), Q30 base percentage in the clean data.

### Differential expression genes analysis

**FIGURE 1:**
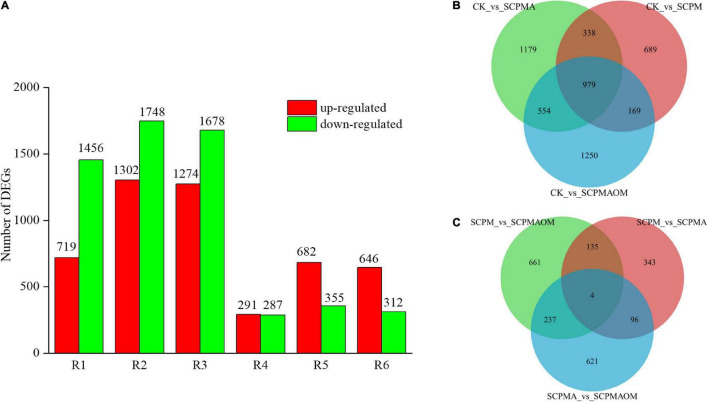
Statistical analysis of the DEGs in different comparisons. **(A)** The number of up/down-regulated genes in different treatments. **(B)** Venn diagram of the DEGs from R1, R2, and R3. **(C)** Venn diagram of the DEGs from R4, R5, and R6. R1, CK vs. SCPM; R2, CK vs. SCPMA; R3, CK vs. SCPMAOM; R4, SCPM vs. SCPMA; R5, SCPM vs. SCPMAOM; R6, SCPMA vs. SCPMAOM.

Large number of DEGs were identified in the comparisons including R1 (CK vs. SCPM), R2 (CK vs. SCPMA), R3 (CK vs. SCPMAOM), R4 (SCPM vs. SCPMA), R5 (SCPM vs. SCPMAOM), R6 (SCPMA vs. SCPMAOM). As shown in Figure 1A, the number of identified DEGs were as follows: R1 (719 up- and 1456 down-), R2 (1302 up- and 1748 down-), R3 (1274 up- and 1678 down-), R4 (291 up- and 287 down-), R5 (682 up- and 355 down-), R6 (646 up- and 312 down-). Venn diagram was performed to analyze the DEGs distribution in comparison group 1 (R1, R2, R3) (Figure 1B) and group 2 (R4, R5, R6) (Figure 1C). The DEGs of R2 (CK vs. SCPMA) and R6 (SCPM vs. SCPMAOM) occupied the largest proportion of total DEGs in comparison group 1 and group 2, respectively. Moreover, the shared DEGs were 979 and 4 in comparison group 1 and group 2, respectively (Figures 1B,C). To evaluate the repeatability of biological repeats, the correlations among different treatments were analyzed. The results showed that the correlation coefficients among three biological repeats in the same treatments were all more than 0.96, which were significantly higher than those among samples from different treatments (Supplementary Figure 2). Furthermore, principal component analysis showed good intragroup correlation and high repeatability (Supplementary Figure 3), suggesting the samples were reliable for use in subsequent data analysis.

### Gene ontology and kyoto encyclopedia of genes and genomes annotation of differentially expressed genes

**FIGURE 2:**
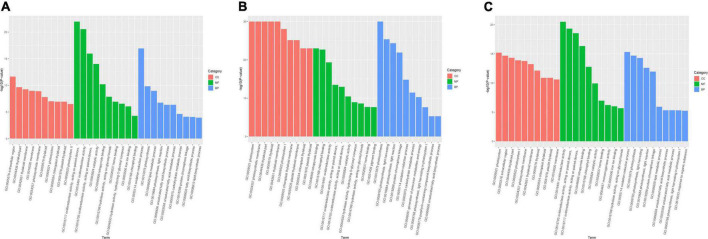
Histogram of GO classification. **(A)** DEGs in R1 comparison, **(B)** DEGs in R2 comparison, **(C)** DEGs in R3 comparison. The DEGs were annotated into three main categories: biological process, cellular component and molecular function. R1, CK vs. SCPM; R2, CK vs. SCPMA; R3, CK vs. SCPMAOM.

Gene ontology classification was performed to annotate DEGs in different comparisons (R1, R2, R3) (Figure 2). For each comparison, top 10 enriched terms in the categories of cell component (CC), molecular function (MF), and biologic process (BP) were selected for further analysis. In comparison R1, the significantly enriched terms in “CC” were, ‘photosystem’ (20 DEGs), ‘extracellular region’ (39 DEGs), ‘thylakoid part’ (32 DEGs), ‘photosystem I’ (11 DEGs). The major terms of “MF” included ‘oxidoreductase activity’ (171 DEGs), ‘oxidoreductase activity, acting on paired donors’ (52 DEGs), ‘tetrapyrrole binding’ (48 DEGs). For “BP,” the DEGs were significantly classified into terms including ‘oxidation-reduction process’ (144 DEGs), ‘photosynthesis’ (31 DEGs), and ‘protein-chromophore linkage’ (12 DEGs).

**FIGURE 3:**
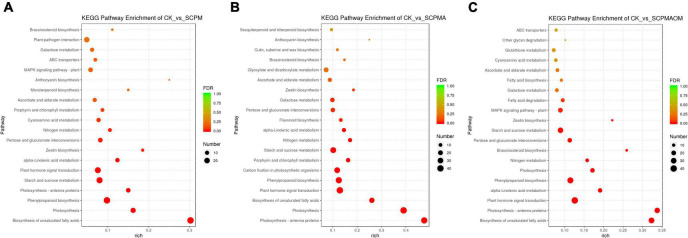
The KEGG pathways enrichment analysis of DEGs from different comparisons. **(A)** KEGG pathways enriched in R1 comparison. **(B)** KEGG pathways enriched in R2 comparison. **(C)** KEGG pathways enriched in R3 comparison. R1, CK vs. SCPM; R2, CK vs. SCPMA; R3, CK vs. SCPMAOM.

KEGG enrichment analysis was conducted to classify the DEGs from comparisons R1, R2 and R3 into 20 significant pathways (*P* < 0.05). In R1, the main enriched pathways were ‘biosynthesis of unsaturated fatty acids’ (29 DEGs), ‘photosynthesis’ (17 DEGs), ‘phenylpropanoid biosynthesis’ (29 DEGs), ‘photosynthesis-antenna proteins’ (12 DEGs), ‘starch and sucrose metabolism’ (25 DEGs), and ‘plant hormone signal transduction’ (Figure 3A). For R2, the remarkably enriched pathways were ‘photosynthesis-antenna proteins’ (38 DEGs), ‘photosynthesis’ (41 DEGs), ‘biosynthesis of unsaturated fatty acids’ (25 DEGs), and ‘carbon fixation in photosynthetic organisms’ (37 DEGs) (Figure 3B). The DEGs of R3 were mainly involved in ‘biosynthesis of unsaturated fatty acids’ (31 DEGs), ‘photosynthesis-antenna proteins’ (27 DEGs), ‘α-linolenic acid metabolism’ (17 DEGs), and ‘phenylpropanoid biosynthesis’ (DEGs) (Figure 3C). In the comparisons of R1, R2, and R3, the up-regulated DEGs were mainly enriched in the pathways like ‘photosynthesis,’ ‘photosynthesis-antenna proteins,’ and ‘plant hormone signal transduction,’ while the down-regulated DEGs were mainly associated with the pathways including ‘biosynthesis of unsaturated fatty acids,’ ‘phenylpropanoid biosynthesis,’ and ‘starch and sucrose metabolism.’ The details of GO and KEGG annotation in these comparisons were shown in Supplementary Table 2.

### Differentially expressed genes related to photosynthesis pathway

**FIGURE 4:**
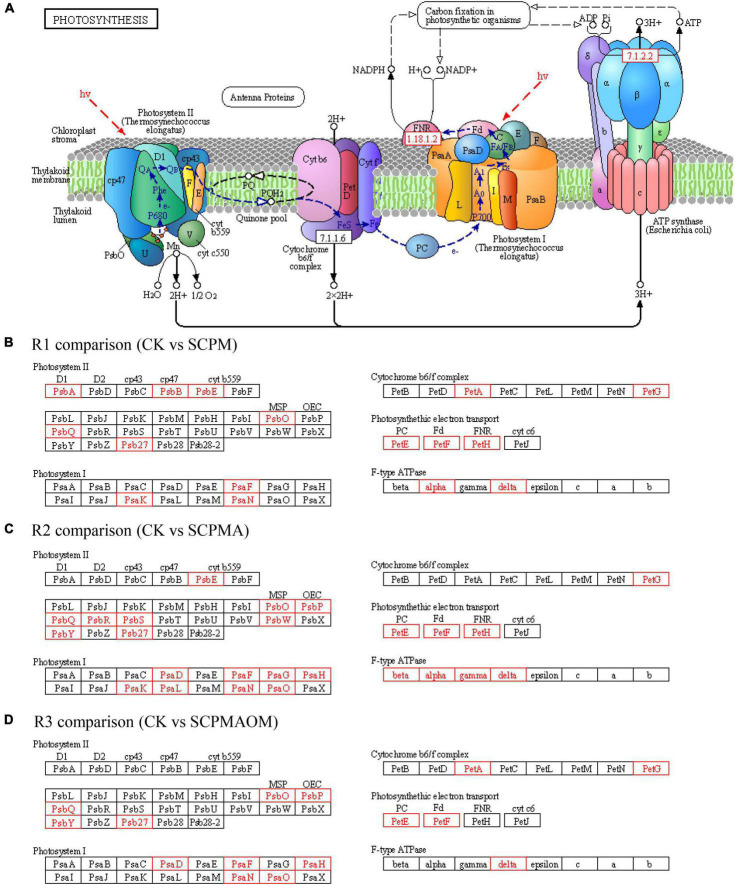
Differentially expressed genes (DEGs) associated with photosynthesis pathway **(A)** in R1 **(B)**, R2 **(C)**, and R3 **(D)** comparisons. Red frames denoted up-regulated genes, while black frames denoted genes showing no differential expression. R1, CK vs. SCPM; R2, CK vs. SCPMA; R3, CK vs. SCPMAOM.

A majority of DEGs involved in photosynthesis were identified (Figure 4). In the R1 comparison, 16 up-regulated DEGs were involved in the biological process including photosystem II (6 DEGs), cytochrome b6/f complex (2 DEGs), photosynthetic electron transport (3 DEGs), photosystem I (3 DEGs) and F-type ATPase (2 DEGs). The equivalent distribution for other comparisons were as follows: R2 comparison, 9, 1, 3, 8, and 4 DEGs, respectively; R3 comparison, 5, 4, 2, 5, and 1 DEGs, respectively. The genes encoding PsbO, PsbQ, and Psb27 in photosystem II, PsaF and PsaN in photosystem I, as well as PetG in cytochrome b6/f complex, were all up-regulated in the three treatments (SCPM, SCPMA, and SCPMAOA). For photosynthetic electron transport, the genes including PetE, PetF were significantly up-expressed in these three treatments. In addition, the delta gene associated with the critical element F-type ATPase was up-regulated in all the remediation treatments (Figure 4). The results showed that all the DEGs involved in photosynthesis pathway were up-regulated in SCPM, SCPMA and SCPMAOM treatments, compared with the control, thus indicating the significantly up-expressed genes especially the crucial DEGs related to photoreaction and cal-vin cycle might greatly promote the photosynthesis efficiency.

### Differentially expressed genes associated with hormone signal transduction pathway

**FIGURE 5:**
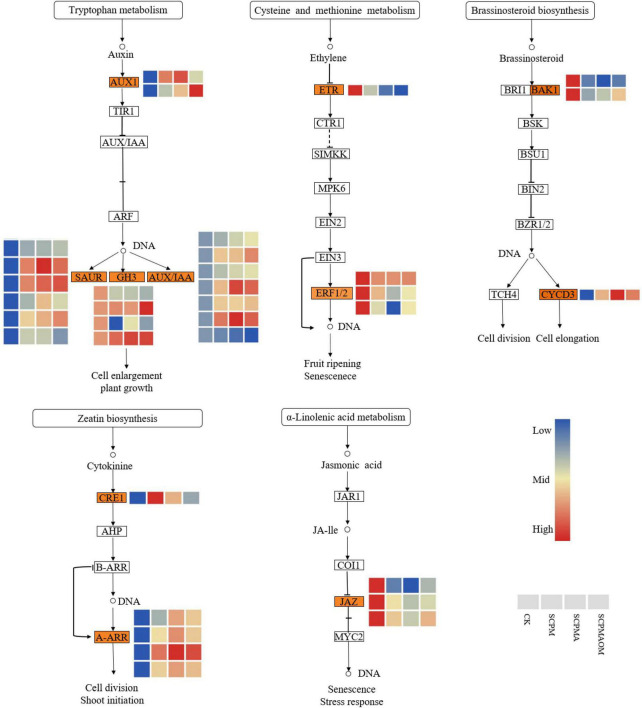
Heat map of DEGs involved in plant hormone signal transduction pathways. Red color denoted genes with high expression level, while blue color denoted genes with low expression level. The relative expression levels of DEGs were calculated using the log2 ratio.

Phytohormones play important roles in plant growth and regulation. Multiple genes related to hormone signal transduction pathway were modulated in all the remediation treatments (SCPM, SCPMA, and SCPMAOM) (Figure 5). The DEGs involved in the pathway of auxin signal transduction, such as *SAUR*, *GH3*, and *AUX*/*IAA*, were significantly up-regulated, compared with the control. In brassinosteroid signal transduction pathway, the *CYCD3* gene was significantly up-regulated. In addition, the *A-ARR* genes associated with cytokinin signal transduction pathway were greatly stimulated in all the treatments. Contrarily, the pathways of ethylene and jasmonic acid signal transductions, were remarkably inhibited in all the remediation treatments. The *ERF1/2* and *JAZ* genes involved in these pathways were significantly down-regulated, thereby slowing down the senescence of *C. tangshen* in the remediation treatments.

### Differentially expressed genes involved in phenylpropanoid biosynthesis pathway

**FIGURE 6:**
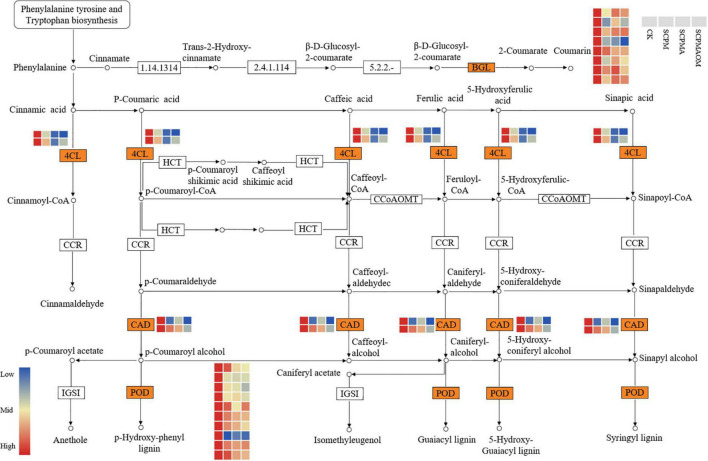
Heat map of DEGs related to phenylpropanoid biosynthesis. Red color denoted genes with high expression level, while blue color denoted genes with low expression level. The relative expression levels of DEGs were calculated using the log2 ratio.

A total of 35 DEGs were annotated into phenylpropanoid biosynthesis pathway. These DEGs were involved in biosynthesis of coumarin, lignin monomers and monoterpenoid (Figure 6). Out of these DEGs, eight genes encoding β-glucosidase (BGL) were remarkably down-regulated in the *C. tangshen* treated with soil amendments. Moreover, 10 genes encoding peroxidase (POD) were down-regulated in the three remediation treatments. Two genes encoding cinnamyl alcohol dehydrogenase (CAD) were significantly down-regulated in all the remediation treatments. Additionally, two genes encoding 4-coumarate–CoA ligase (4CL) were significantly down-regulated in the three remediation treatments, which might indicate that the biosynthesis of lignin was inhibited in the remediation process.

### Differentially expressed genes related to biosynthesis of unsaturated fatty acids

**FIGURE 7:**
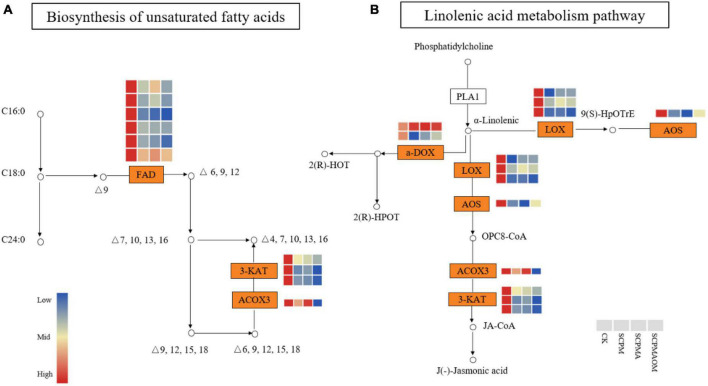
The expression patterns of DEGs in the biosynthetic pathways of unsaturated fatty acid **(A)** and α-linolenic acid **(B)**. Red color denoted genes with high expression level, while blue color denoted genes with low expression level. The relative expression levels of DEGs were calculated using the log2 ratio.

Twenty seven of 29, 23 of 25, and 28 of 31 DEGs involved in unsaturated fatty acids biosynthesis were down-regulated in R1, R2, and R3 comparisons, respectively. The results showed that all the genes encoding fatty acid desaturase (FAD) were significantly down-regulated in all the remediation treatments, compared with the control (Figure 7A). Three genes encoding 3-ketoacyl-CoA thiolase (3-KAT), were significantly down-regulated in the three remediation treatments, and the expression levels were lowest in the SCPMAOM treatment. In addition, one gene encoding acyl-coenzyme A oxidase 3 (ACOX3), which was involved in the first step of fatty acids β-oxidation, was significantly down-regulated in all the treatments. Moreover, the *ACOX3* gene also participated in the pathway of α-linolenic acid metabolism. Additionally, in the jasmonic acid biosynthesis pathway, one gene encoding allene oxide synthase (AOS) was significantly down-regulated in the three remediation treatments, compared with the control. Interestingly, three *3-KAT* genes involved in unsaturated fatty acids biosynthesis pathway were also showed in α-linolenic acid metabolism (Figure 7B). Moreover, the expression levels of three genes encoding linoleate 13S-lipoxygenase (LOX) were significantly lower in the three remediation treatments than those in the control.

### Differentially expressed genes associated with starch and sucrose metabolism

**FIGURE 8:**
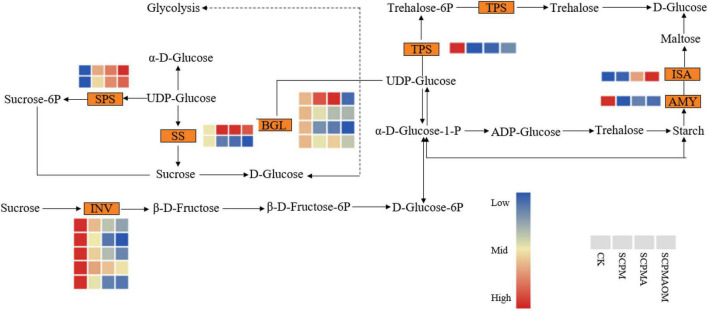
Differentially expressed genes related to the starch and sucrose metabolism pathways in the different treatments. Red color denoted genes with high expression level, while blue color denoted genes with low expression level. The relative expression levels of DEGs were calculated using the log2 ratio.

Multiple genes were significantly enriched in the pathway of starch and sucrose metabolism in *C. tangshen* treated with soil amendments (Figure 8). The results showed that 25, 32, and 28 DEGs were involved in starch and sucrose metabolism in R1, R2, and R3 comparisons, respectively. Generally, α-amylase (AMY) and isoamylase (ISA) participate in the conversion of starch to maltose. *AMY* gene was significantly down-regulated, while *ISA* gene was significantly up-regulated in the SCPMAOM treatment. In the biological process of sucrose metabolism, six genes encoding β-fructofuranosidase (INV) were significantly down-regulated, while two genes encoding sucrose-phosphate synthase (SPS) were significantly up-regulated in the three remediation treatments. Moreover, in the initial stage of UDP-glucose metabolism, four genes encoding β-glucosidase (BGL) and one gene encoding α, α-trehalose-phosphate synthase (TPS) were all down-regulated in the three treatments.

### Validation of the differentially expressed genes by qRT-PCR

**FIGURE 9:**
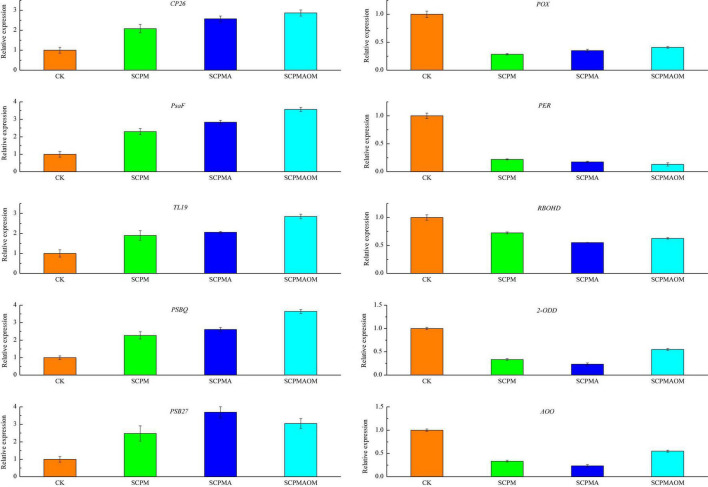
The qRT-PCR analysis of 10 DEGs in the leaves of *C. tangshen* in different treatments. *GAPDH* gene was used as the reference gene for normalization of gene expression level. Three technical replicates were performed.

Ten unigenes responsive to soil remediation were chosen to validate the RNA-Seq results by qRT-PCR (Figure 9). The expression levels of five photosynthesis-related unigenes (*CP26*, *PsaF*, *TL19*, *PsbQ*, and *Psb27*) were significantly increased in the three remediation treatments, while five genes (*POX*, *PER*, *RBOHD*, *2-ODD*, and *AOO*) involved in antioxidant system and redox reaction were remarkably down-regulated in all the remediation treatments. In summary, the expression profiles of these unigenes based on the qRT-PCR were consistent with the RNA-Seq data, indicating the RNA-Seq results were reliable and accurate.

## Discussion

### Physiological and biochemical responses

Continuous cropping obstacle is a serious problem for agricultural production of *C. tangshen* (He et al., 2019; Zhang et al., 2021). In this study, the treatments such as SCPM, SCPMA, and SCPMAOM greatly alleviated the adverse effects of continuous cropping on *C. tangshen*. Photosynthetic pigments, such as chlorophyll a, chlorophyll b and carotenoids are essential for plant growth and development (Kamble et al., 2015). In the present work, the chlorophyll (a, b), total chlorophyll and carotenoid levels were higher in the remediation treatments than those in the control. This was consistent with the results of Zhou et al. (2021), which indicated that organic manure (OM) and silicon–calcium–potassium–magnesium fertilizer (SCPM) could remarkably increase the chlorophyll and carotenoid content in continuously cropped *C. tangshen*. Moreover, the chlorophyll and carotenoid levels were significantly higher in SCPMAOM than those in CK, SCPM, and SCPMA, indicating that silicon-calcium-potassium-magnesium fertilizer, azoxystroxbin and organic manure have synergistic effects on photosynthetic metabolism in *C. tangshen*.

Antioxidant enzymes, e.g., SOD, POD and CAT, are important proteins related to the protection of the plasma membrane through prevention of peroxidation in plants (Gill and Tuteja, 2010; Kapoor et al., 2019). In this study, the significantly decreased SOD and slightly increased POD activities were occurred in the three remediation treatments (SCPM, SCPMA, and SCPMAOM), and the CAT activity was significantly increased in SCPMA and SCPMAOM treatments, which was basically consistent with previous study (Zhou et al., 2021). These results suggested the increased POD and CAT activities might be involved in the prevention of peroxidation in *C. tangshen*. Comprehensively, the levels of yield and alkaloid were significantly increased in the three remediation treatments, and the yield of SCPMAOM treatment was 10 times higher than that of the control. Moreover, the levels of polysaccharide, flavone, alkaloid and total protein were all increased in the three remediation treatments and these indexes reached significant level (*p* < 0.05) in SCPMAOM treatment. This indicating that the comprehensive stimulative effects on yield and quality (polysaccharide, flavone, alkaloid, and total protein) followed the order of SCPMAOM > SCPMA > SCPM > CK, which further certificated that silicon-calcium-potassium-magnesium fertilizer, azoxystroxbin and organic manure had cumulative effects on the growth and development of *C. tangshen* under continuous cropping system.

### Differentially expressed genes related to photosynthesis

Photosynthetic apparatuses represented by photosystem II, cytochrome b6/f complex, photosystem I and F-type ATPase are easily influenced by environmental changes (Dey et al., 2022). Numerous studies in the past decades have demonstrated that the extrinsic PsbO, PsbP, and PsbQ proteins play important roles in maintaining optimal manganese, calcium and chloride concentrations at the active site of photosystem II, and chemical or genetic removal of these components will induce multiple and profound defects in photosystem II function and oxygen-evolving complex stability (Bricker and Frankel, 2011; Ifuku and Nagao, 2021). In this study, the *PsbO*, *PsbP*, and *PsbQ* genes in *C. tangshen* were significantly up-regulated in all the remediation treatments, indicating that the soil amendments might promote the photosynthetic metabolism of *C. tangshen* by enhancing the functions of photosystem II. Similarly, the significantly up-regulated genes of *PsaF* and *PsaN* in photosystem I might also play a pivotal role in stimulating the photosynthesis of *C. tangshen* under remediation system.

Cytochrome b6f complex is an enzyme found in plants, cyanobacteria, and green algae which catalyzes the electron transport in the rate-limiting step of oxygenic photosynthesis (Mehling, 2020). The results showed that the *PetA* and *PetG* genes were greatly up-regulated in both SCPM and SCPMAOM treatments, demonstrating that *PetA* and *PetG* might play a key role in the electron transfer involved in the photosynthesis of *C. tangshen* under remediation system. F-type ATPase, which resides in the chloroplast thylakoid membranes, can transform ADP into ATP and provide energy for carbon fixation in photosynthetic organism (Kühlbrandt, 2019). The results showed that ATPase components including beta, alpha, gamma, and delta subunits were integrated and activated in the ATP synthesis pathway, indicating plenty of ATP was synthesized, which contributed to the carbon fixation in photosynthetic organism in *C. tangshen* during the soil remediation. Interestingly, the enhanced expressions of genes involved in photosynthetic metabolism were consistent with the increased chlorophyll and carotenoid levels in soil remediation treatments, indicating that the soil amendments might promote the growth of continuously cropped *C. tangshen* by improvement of photosynthesis.

### Differentially expressed genes associated with plant hormone signal transduction

Plant hormones are always associated with the regulation of growth and environmental adaptability in higher plants (Kou et al., 2021). Auxin (IAA) plays a key role in regulating plant growth, development and response to environmental stresses (Liu et al., 2014; Singh et al., 2021). In this study, most of the genes involved in IAA signal transduction pathway, such as *SAUR*, *GH3*, and *AUX1*, were significantly up-regulated in the soil remediation treatments, thereby stimulating the cell enlargement and plant growth. This result was in accordance with the higher yield in the soil remediation treatments (Table 3). Brassinosteroids (BRs) are steroid hormones which are essential for plant growth and development. These hormones control the division, elongation and differentiation of various cell types throughout the entire plant life cycle (Planas-Riverola et al., 2019). The *D-type cyclin* (*CYCD*) gene family plays an important role in promoting cell division in BR signal transduction (Dewitte et al., 2007). In our study, the expression levels of *CYCD3* genes were significantly higher in the soil remediation treatments than those in the control, which might improve the mitotic activity in cambium. Hence, it was comprehensible that the stronger roots and higher yields of *C. tangshen* were observed in SCPM, SCPMA, and SCPMAOM treatments, compared with the control (Table 3).

The phytohormone cytokinin (CK) plays diverse roles in plant development, cell division and shoot initiation (Kieber and Schaller, 2018). In our study, the expression levels of *CRE1* and *A-ARR* genes involved in the CK signal transduction were significantly increased by all the soil remediation treatments, which might finally promote the cell division and shoot meristem initiation in *C. tangshen*. Ethylene-responsive transcription factors (ERFs) play important roles in tolerance to biotic and abiotic stresses by regulating the expression of stress-responsive genes (Wu et al., 2014). The expressions of *ERF1/2* genes were greatly decreased by all the remediation treatments, indicating that the defensive response was not completely activated due to the slighter stress in the soil remediation treatments. Jasmonic acid (JA) plays important roles in plant growth and development, especially plant responses to biotic and abiotic stresses (Ruan et al., 2019). In the present work, the *JAZ* genes involved in the JA signal transduction pathway were significantly down-regulated in the soil remediation treatments, indicating that the soil remediation measures might alleviate the continuous cropping induced stress in *C. tangshen*, which resulting in the down-regulation of *JAZ* genes in remediation system.

### Differentially expressed genes involved in phenylpropanoid biosynthesis

Reportedly, the phenylpropanoid biosynthesis is usually involved in defense metabolisms (Yadav et al., 2020). For instance, some phenylpropanoids are important substrates for the synthesis of lignins and play key roles as protectants, phytoalexins and antioxidants in most of the higher plants (Deng and Lu, 2017). The genes associated with lignin, coumarin, and monoterpenoid biosynthesis, such as *BGL*, *4CL*, and *CAD*, were involved in the biological responses to various stresses (Krithika et al., 2015; Baba et al., 2017; Qiu et al., 2018). In this study, most of the genes encoding BGL, 4CL, and CAD were significantly down-regulated in the soil remediation treatments, indicating that the *C. tangshen* in remediation treatments were grown in a better environmental condition than those in the control. Peroxidase (POD) is involved in antioxidant activity and cell wall lignification, which protect plants from damages induced by various stress (Lee et al., 2021). Previous studies showed that greatly expressed POD genes were found involved in antioxidant activity and lignin synthesis in chestnut and tea plants (Cao et al., 2020; Wu et al., 2022). In this study, most of the *POD* genes were significantly down-regulated in the three soil remediation treatments (SCPM, SCPMA, and SCPMAOM), which again validated the functions of soil amendments in alleviating the continuous cropping induced stress.

### Differentially expressed genes related to unsaturated fatty acid metabolism

Some fatty acids play critical roles in plant defense against abiotic stress conditions (Begara-Morales et al., 2021). Previous studies showed that unsaturated fatty acids were closely related to membrane stability, and positively correlated with the degree of environmental stress (Liu et al., 2011; Chen et al., 2022). Fatty acid desaturase 2 (FAD2) catalyses oleic acid (OA) to linoleic acid (LA) in the second step of fatty acid desaturation pathway (Xue et al., 2017; Fan et al., 2019). In this study, all the *FAD2* genes were down-regulated in the three remediation treatments, which might lead to the decreased biosynthesis of unsaturated fatty acids, indicating that the abiotic stress in *C. tangshen* were mitigated by soil remediation treatments. In α-linolenic acid metabolism, acyl-coenzyme A oxidase 3 (ACOX3) and 3-ketoacyl-CoA thiolase (3-KAT) were involved in the first and second step of fatty acid β-oxidative cracking reactions, respectively (Froman et al., 2000; Sundaramoorthy et al., 2006). Our study showed that all the *ACOX3* and *3-KAT* genes were significantly down-regulated in the three remediation treatments, and the lowest expressions were observed in SCPMAOM treatment, indicating less α-linolenic acids were accumulated in SCPMAOM treatment. Thus, we speculated that SCPMAOM showed the best remediation effects on the continuous cropping soil.

### Differentially expressed genes engaged in starch and sucrose metabolism

α-amylase (AMY) was involved in starch conversion into dextrins and fermentable sugars in crops like corn, barley, potato, or rice (Li et al., 2022), and isoamylase (ISA) could hydrolyze α-1,6-glucosidic linkages in amylopectin to yield amylose and oligosaccharides (Li et al., 2017b). In this study, the *AMY* gene was significantly down-regulated in the three remediation treatments, which suggested the degradation of starch might be reduced and the accumulation of starch promoted the growth and development of *C. tangshen* in remediation treatments. However, the *ISA* expression change was inconsistent with that of *AMY*, suggesting the complexity of starch metabolism in *C. tangshen* and further verification was required. In sucrose metabolism, β-fructofuranosidase (INV) possesses the transfructosylation activity of transferring sucrose into fructose and glucose, while sucrose-phosphate synthase (SPS) is related to sucrose synthesis (Anur et al., 2020). The results showed that the *INV* genes were significantly down-regulated, while the *SPS* genes were significantly up-regulated in the SCPM, SCPMA, and SCPMAOM treatments, indicating the degradation of sucrose was inhibited and the sucrose biosynthesis was stimulated, which might gradually promote the growth and development of *C. tangshen* in remediation treatments. Additionally, the highest expression of *SPS* and the lowest expression of *INV* were observed in the SCPMAOM treatment, which again validated the best remediation effects of SCPMAOM.

## Conclusion

**FIGURE 10:**
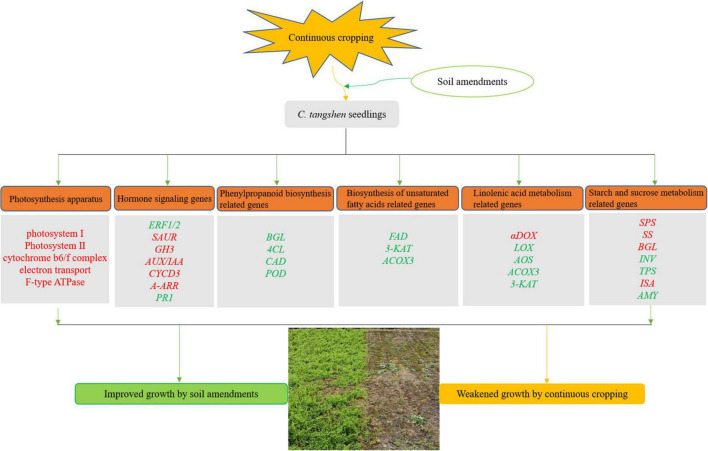
A hypothetical model of soil amendments induced response in continuously cropped *C. tangshen*. The apparatus or genes labeled with red were up regulated, while labeled with green were down regulated.

This study firstly demonstrated how continuously cropped *C. tangshen* responded to soil amendments at the transcriptional level. The yield and quality was improved by the remediation treatments, and the SCPMAOM treatment (3000 kg/hm^2^ silicon-calcium-potassium-magnesium fertilizer combined with 500 times azoxystrobin solution and 4500 kg/hm^2^ organic manure) showed the best remediation effects on the continuous cropping soil. The results revealed that the soil amendments improved the tolerance of *C. tangshen* to continuous cropping by regulating the genes involved in pathways like ‘photosynthesis,’ ‘photosynthesis-antenna proteins,’ ‘plant hormone signal transduction,’ ‘phenylpropanoid biosynthesis,’ ‘biosynthesis of unsaturated fatty acids,’ and ‘starch and sucrose metabolism’ (Figure 10). The synergies of these pathways improved the growth and development of continuously cropped *C. tangshen* in soil remediation treatments. This study was beneficial for unraveling the molecular mechanisms of soil amendments in regulating the growth of continuously cropped *C. tangshen* at transcriptome level and provided a technical support for improving the tolerance of *C. tangshen* to continuous cropping.

## Data availability statement

The original contributions presented in this study are publicly available. This data can be found here: https://ngdc.cncb.ac.cn/, CRA006788.

## Author contributions

WZ and XJ designed the experiment and completed the manuscript writing. XT and JY participated in the experimental design. WZ, XJ, DL, HW, and XL carried out the experiment and processed the experimental data. MZ reviewed and finalized the manuscript. All authors reviewed and approved the manuscript.

## Supplementary material

Supplementary Figure 1 Leaf morphology of *Codonopsis tangshen*.

Supplementary Figure 2 Correlation analysis of gene expression profiles.

Supplementary Figure 3 The principal component analysis (PCA) of gene expression profiles.

Supplementary Table 1 The information of primers used for qRT-PCR.

Supplementary Table 2 Go and KEGG annotation of CK vs SCPM, CK vs SCPMA, and CK vs SCPMAOM comparisons.
