# Supplementary figures and images for: Transcriptome analysis provides novel insights into the soil amendments induced response in continuously cropped Codonopsis tangshen
Source: Front Plant Sci. 2022 Aug 12;13:972804. doi: 10.3389/fpls.2022.972804 (PMC9413139; doi:10.3389/fpls.2022.972804)

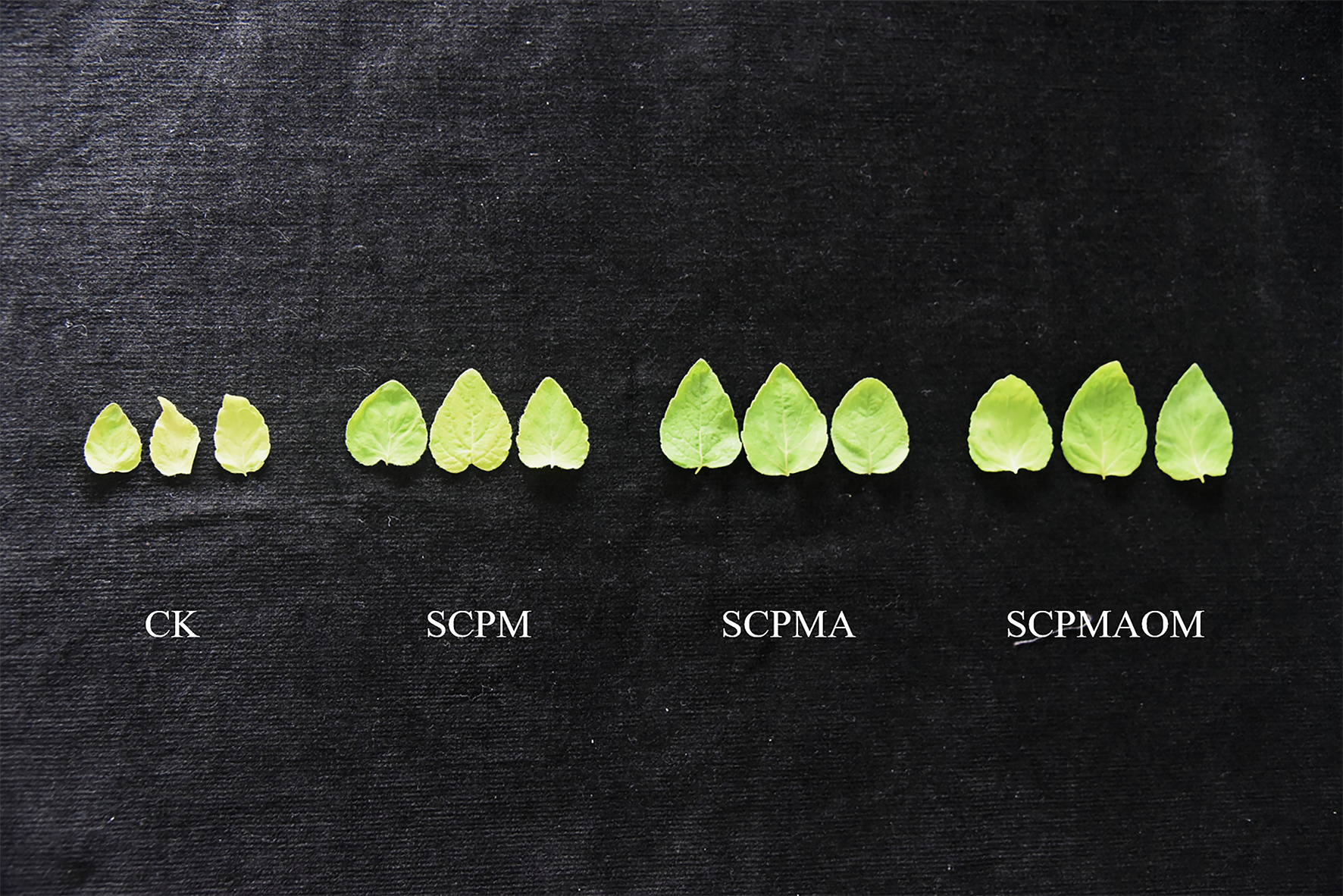

Supplement: Supplementary Figure 1 — Leaf morphology of Codonopsis tangshen. [file Image_1.TIF]

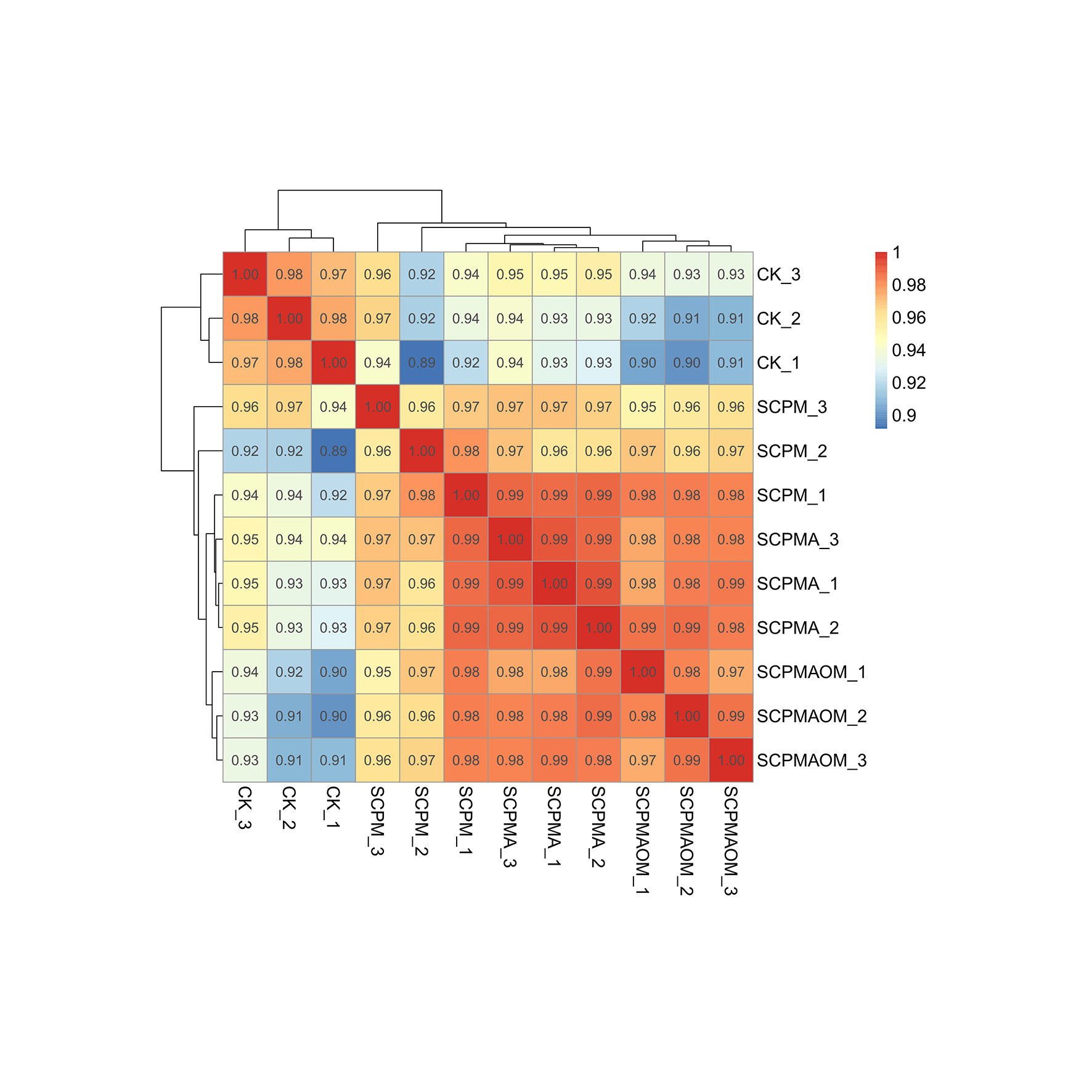

Supplement: Supplementary Figure 2 — Correlation analysis of gene expression profiles. [file Image_2.JPEG]

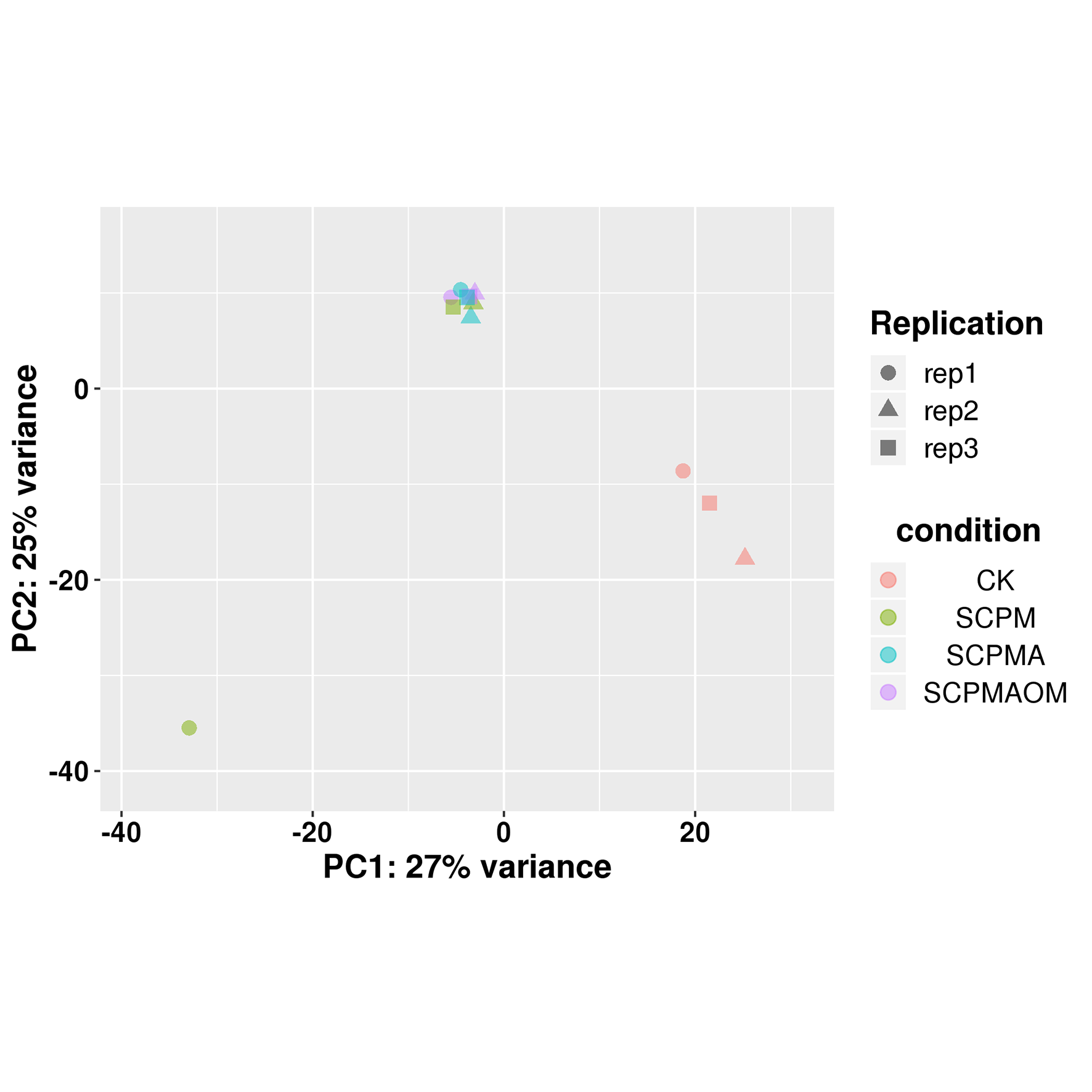

Supplement: Supplementary Figure 3 — The principal component analysis (PCA) of gene expression profiles. [file Image_3.PNG]
